# Letter from London

**Published:** 1890-08

**Authors:** E. J. Beall

**Affiliations:** No. 37 Bedford Place, Russell Square, W. C., London


### Correspondence.

### LETTER FROM LONDON--SPARKLING AND NEWSY.

No. 37 Bedford Place, Russell Square, W. C.,
London, July 30, 1890.

*Editor Daniel's Texas Medical Journal:*

Needing rest from professional work, I left home early in June for this English speaking metropolis, with her many old and new colleges and hospitals.

At New York I lingered a week, renewing associations of the years that have passed into the night of time and recalling memories that go back to student life under Mott (the elder), and Payne, Swett, Van Buren, Draper, and Markoe. Alas! of all this galaxy only the venerable Markoe remains. I am here reminded of a pleasant remark which came from Prof. Markoe. Meeting him at the Surgical Society I said to him that I had heard him him lecture upon bone pathology, at the University of New York, in 1855. He replied, "Doctor, you are mistaken." "No," said I, "often have I seen you come in with your *bag of bones*, which, perhaps, now forms part of a New York museum as specimens in pathological osteology." "Yes, yes," he said,

"I do recall the fact that I lectured on bone pathology at the University that year; but *don't* tell the girls!"

I enjoyed myself very much, socially and professionally, while in New York. And let me here remark that as good work can be found in that city as in this. Of this there is no question in my mind. And why not? While the English curriculum is long, greater care is taken in teaching, etc., and doctors are not pitched out of the college doors after so short a probation, and in such great numbers, yet, in America, I know, that we have men in the profession whose student days do not end with their college terms, but they remain students as long as in the professional harness. Such men will not be restrained by the shortcomings of teaching, but will, and do, forge to the front, attain efficiency and do as good work as men of any other country. Why not? It cannot be said that we have no native intellect; that our beautiful, healthful country with its push and vim, and freedom of thought and action make men pigmies. Away with such a paradox—such heterodoxy!

I took dinner with Prof. Gibney, and had a drive through the park with him. Gibney is one of America's foremost orthopedic surgeons, and although he has sought and enjoys a larger field for his brain and work than his home offered, the Kentucky gentleman clings to him,—he has that homelike hospitality and fondness for horses that tell of his nativity.

We spent a pleasant evening at the home of the affable Wyeth. Just now a sadness broods over his pleasant home; for at his delightful dinner table I missed a face and form, saw a vacant chair—Mrs. J. Marion Sims had died since I last sat in his dining room. The good wife, the queenly woman, rests by the side of one of the best friends of womankind, and if we measure greatness by the good that follows one's life, by the work that outlives the worker, Sims was an American genius—the greatest of American men. Here, in the old world, at every turn and corner, statues and monuments are seen perpetuating the life and example of great men. America, the Southland, the country of his birth, should to Marion Sims raise a monument so high that a star would crown its pinnacle and the sun of another world than ours illumine and reflect its glories.

I very much enjoyed the surgical work I saw in New York, done by Wyeth, Bull, Wylie, Hartly, Abbey, Gibney, Sims and others. Wyeth did a new operation, different from the procedure of Langenbeck and others, for restoring a nose which years before had been driven in at the bridge. He made a subcutaneous division of the bone down the center of the depression; then a subcutaneous division of either superior-maxilla, just avoiding the duct to the nose, then with chisel and wrapped forceps loosened the fragments he had made, elevated them with Sayer's elevator introduced into the nostrils, and fastened them while in this elevated position by passing steel spikes horizontally through them; over this antiseptic dressing. Before I left New York a good result was evident.

There was nothing new in the laparatomies I witnessed; all such operations having been done quickly and expeditiously. Two were for salpingitis, one for an ovarian cyst. In one case, operated on by Sims, there was a very clear demonstration of the condition Wylie claims to be at the bottom of all pelvic abscesses: that the abscess is due to perforation of the tube—to salpingitis.

Thiersch's operation of skin-grafting I saw done, and examined a case or so which promised good results—the operation having been performed some days before I reached New York. It is claimed by some that results cannot prove good after Thiersch's method, that there will not be stability and permanence to the graft, but that it is early destroyed by ulcerative processes even after one concludes good results are secured. The cases I saw were after removal of the breast. Taking the opinions of the many the method perhaps is not well established as a surgical procedure.

I arrived at Liverpool the Wednesday after sailing from New York upon steamship City of York, eight days on the Atlantic. The trip was fairly good, yet many of the twelve hundred passengers experienced the *Mal de Mer*. I was not sick and very much enjoyed the daily professional conversations participated in by such men as Hermann Knapp, of New York, Chisholm, and "Help for poor crippled women" H. P. C. Wilson, the jovial, kind and able gynecologist of Baltimore. Dr. H. K. Leake, of Dallas, was also with us. By way of parenthesis I must tell you the origin of "Shirt-tail Poker." On one occasion four of the most distinguished gynecologists of America were guests of one of the above party in Baltimore. After an elegant dinner and all the toasts were passed, and all the participants had retired—at one or two a. m. a game of poker was suggested. The distinguished gentlemen herein referred to arose from their respective beds, and, all in "undress uniform," began a game which only ended with the darkness of the night. Hence, the term "Shirt-tail Poker." This information I first heard between continents—upon the wide Atlantic.

I loitered one day at Chester, visited the Castle of Westminster; the old church, built A. D. 698, during the reign of King Ethelred, the Saxon; the walls built some centuries before upon the old Roman walls; then went to Shrewsbury and saw the castle in which Falstaff fought four hours; ran down to Stafford-on-Avon, visited the house where Shakspeare was born; then the church and stood over the grave of him of whom it has been said that he was "in judgment a Newton, in intellect a Socrates, in art a Virgil. The earth covers, the people mourn, Olympus has him."

I brought away a piece of the floor over which Shakspeare walked and which was put down two hundred years before his birth. The epitaph upon his tombstone is very plain. This you recollect; and it is because of it that his bones still lie in this old church of the thirteenth century (which doubtless occupies the site of a more ancient building,) instead of being placed in Westminster Abbey.

For more than three weeks I have been in the British metropolis, and although historic in every direction; the home of kings; abounding in relics of wars which antedate the discovery of our beloved continent, that discovery commemorated by statues in gilt and marble—yet, the old and new hospitals hold the interest that attract me daily and hourly. To think that I daily walk the steps once trodden by John Hunter, Sir Astley Cooper, Abernathy, Harvey, and a host of grand old men, giants indeed in intellect, it seems that I get an inspiration at Kings, St. Bartholmew's, Guy's and other places that makes it pleasant to be here.

Since my arrival I have seen numbers of the great living men of this largest city of the world, and have been an attentive witness at the operating table and in the hospital ward.

I have been very much pleased with many of the profession whom I have met here, and with the work they have done which has made their names household words with the profession in every civilized country. I cannot see, though, that the profession here are in advance of our best men in America. In fact, they are behind in some respects. The surgeons here are about equally divided in the use of ether and chloroform. At the Soho hospital for women I saw nitrous-oxide gas used in the beginning of anæsthesia and the influence kept up by ether. This was in the service of Dr. Harry Fenwick. In all the operations I have witnessed outside of the Royal Opthalmic, Moorfield's, I have not seen cocaine anæsthesia resorted to in a single instance. At St. Peters, for genito-urinary, the largest clinic I ever saw, I was surprised to see ether and chloroform used in cases in which we usually resort to cocaine. Much time is wasted by the non-usage of local anæsthesia, to say nothing of the difference in danger, as compared with general anæsthesia with ether and chloroform. The mixture of ether and chloroform (Vienna mixture) I saw used several times.

I was surprised to see the Simon speculum, or a short-bladed Sims used—the patient placed upon the back—in several cases of gynecology.

The use of the Esmarch bandage is obsolete here—is not being used at all; and Mr. Heath told me that he doubted if an Esmarch could be found in a London hospital. He showed me a case in the person of a girl whose knee-joint had been resected, in whom, though she had perfect recovery from the operation, paralysis was present, and this condition was considered due to the Esmarch bandage. I heard Sir Joseph Lister, Bart., say in the course of his remarks about a resection of the elbow he was then doing: "That the pressure of the Esmarch bandage upon the nerves, forcing and retaining them against the humerus, tended to induce paralysis; hence, he had discarded the appliance from his practice."

Speaking of Sir Joseph Lister reminds me of his extreme care in regard to the antiseptic procedure in all the operations I have seen him do. We are familiar with his mode, and do in America as he does here, and as those who follow clean surgery elsewhere do. Upon the whole I do not observe as strict attention to strict antisepsis here as I did in New York. Many, like Mr. Ed. Owen, Mr. Sidney Rodgers, Mr. Fred. Trives, and others, carry out the principle to a certain degree, and do rapid work—closing wounds quickly.

At Great Ormand St. hospital I have seen Mr. Owen operate, and have gone through his surgical wards. In osteotomies he prefers the saw to the chisel, and uses Thomas' splint for fixation. I am pleased with his operation and his preferences over those of my esteemed friend Gibney, of the New York Orthopedic hospital, who seems to prefer the chisel and plaster fixation.

The disposition of the most of the surgeons here is to use drainage after operations. I noticed an exception in the practice of Mr. Barker, at University College hospital. I saw him excise hip-joint, curette the acetabulum and disease of the soft parts, wash the cavity with emulsion of iodoform and seal up thoroughly with sutures. Also saw him freely incise a psoas abscess, curette the abscess cavity, wash with emulsion of iodoform and close. He has had quite a succession of good results in a number of these formidable cases.

I brought over to this country Prof. Wyeth's method of bloodless operation at the hip-joint, which, the author thinks, will reduce the mortality to four per cent. Dr. Treves will notice this in his work on operative surgery which is being prepared for the press at this time, and which I predict will be one of the most valuable works of the kind that has been before the profession. At St. Thomas', in a hip-joint amputation, compression was done only at Poupart's ligament, and after the anterior cut through the skin and fascia was made, the femoral was isolated and tied, the soft parts divided down to the trochanter, then the gluteal and other branches tied as divided. No great amount of blood was lost.

There is a tendency here to use small instruments. Catlings are no longer used in amputations, and all small vessels are controlled as the work proceeds, thus limiting the loss of blood.

I am very much pleased with Mr. Bantock, at the Samaritan.

I have seen him in several abdominal sections. His work is nice, clean and good. I very much admire his plan of dressing the abdomen; it is novel, and I like it better than that of any operator I have seen.

Sir Spencer Wells is not doing any hospital work now, and is perhaps limiting his cases to a few a week. I called and was kindly received by this pleasant, jovial Nestor of abdominal surgeons; and hope I shall have an opportunity to see him operate before I return to America.

I am very much in love with the mode of teaching in London. It is far superior to ours, or at least to that of most of the colleges in America. There is a tendency here to very much limit didactic lectures, and to give very much more attention to clinical work.

In giving attention to internal medicine, to the skin, eye, etc., we go with the teacher through the wards, the student is brought face to face with the case, and the case is studied at the bedside. I was so much pleased with Dr. Barlow and Dr. Green in this line of work. The skin clinics are large. Eczemas, tinæ tonsurans, and lupus can certainly be thoroughly studied here. At London rodent ulcer can be produced in numbers, and the disease studied in its various stages. The same may be said of the eye. At the Royal Opthalmic hospital, Moorfield, perhaps the largest eye clinic in the world, one finds a daily feast from nine a. m. until three o'clock p. m.; case after case, operation after operation.

There seems to be a split in the profession here that will militate against the British Medical Association. It is a jealousy or like feeling, which exists between the provincial and London profession. I am almost disuaded from losing time in attending that meeting.

The French, I fear, will not support the Congress as they should—a conflicting Congress having been organized. I hope, however, to meet and see some of their best workers at Berlin—don't know how they can keep away. I find that in London the feeling is favorable to the Congress; and that many will let the British Medical Association go and attend the Congress.

In another week I shall leave for the continent. I wish to see

Pean, Doléris, and Apostoli, and to spend some days in their respective clinics. I shall visit the hospitals at Cologne; Geneva, to see Cordes; thence to Munich and Dresden, winding up at Berlin, to gain what I can with my eyes at Martin's, Von Bergmann's, and the Victoria clinics. Will return to London for three weeks study; spend a day at each hospital in Edinburgh, Glasgow and Dublin, sailing for home in September. Then a week each at New York and Philadelphia, a day in Baltimore with Dr. Chisholm and Dr. Wilson, and the Johns Hopkins, to which I have been invited; a day at the Washington hospitals, and a day at the city and the St. John's hospitals, St. Louis; then for dear home and Texas. Yours truly,

E. J. BEALL, M. D.

